# Speckle-Tracking Analysis of the Right and Left Heart after Peak Exercise in Healthy Subjects with Type 1 Diabetes: An Explorative Analysis of the AppEx Trial

**DOI:** 10.3390/jcdd10110467

**Published:** 2023-11-17

**Authors:** Paul Zimmermann, Janis Schierbauer, Niklas Kopf, Harald Sourij, Nick Oliver, Felix Aberer, Nadine B. Wachsmuth, Othmar Moser

**Affiliations:** 1 Division of Exercise Physiology and Metabolism, BaySpo—Bayreuth Center of Sport Science, University of Bayreuth, 95440 Bayreuth, Germany; paul.zimmermann@arcormail.de (P.Z.); janis.schierbauer@uni-bayreuth.de (J.S.); niklas.kopf@uni-bayreuth.de (N.K.); nadine.wachsmuth@uni-bayreuth.de (N.B.W.); 2 Interdisciplinary Center of Sportsmedicine Bamberg, Klinikum Bamberg, 96049 Bamberg, Germany; 3 Department of Cardiology, Klinikum Bamberg, 96049 Bamberg, Germany; 4 Interdisciplinary Metabolic Medicine Research Group, Division of Endocrinology and Diabetology, Medical University of Graz, 8036 Graz, Austria; ha.sourij@medunigraz.at (H.S.); felix.aberer@medunigraz.at (F.A.); 5 Faculty of Medicine, Department of Metabolism, Digestion and Reproduction, Imperial College London, London SW7 2AZ, UK; nick.oliver@imperial.ac.uk

**Keywords:** echocardiography, strain analysis, type 1 diabetes, cardiac remodeling, diabetic cardiomyopathy

## Abstract

In eight healthy participants with Type 1 diabetes (T1D) exercise-related dynamic cardiac remodeling was analyzed by performing two-dimensional echocardiography, including deformation analysis of the left-ventricular (LV) global longitudinal strain (LV-GLS), and the deformation pattern of the left atrium (LA) and right ventricle (RV) at rest and post-peak performance on a bicycle. The feasibility echocardiographic speckle-tracking analysis was performed on eight asymptomatic participants with T1D (*n* = 8, male *n* = 5, age: 23–65 years). The obtained echocardiographic data were compared for various echocardiographic parameters at rest and post exercise. Across our participating T1D individuals no structural echocardiographic abnormalities of concern could be revealed. All participating T1D subjects showed preserved contractile reserve of the LV and no significant diastolic dysfunction. Significant differences were found for the phasic LA contractile strain pattern at rest and post exercise (*p* < 0.001), whereby the dynamic RV (*p* = 0.5839 and *p* = 0.7419) and LV strain pattern (*p* = 0.5952) did not reveal significant differences in comparison to resting conditions. This descriptive secondary outcome analysis describes preserved contractile reserve of the LV and elucidates dynamic modification of the phasic LA contractile deformation pattern in asymptomatic T1D individuals after exhaustive exercise on a bicycle.

## 1. Introduction

The prevalence of diabetes mellitus is increasing rapidly and is estimated to affect 300 million adults in 2025 [1]. Type I diabetes (T1D) and Type 2 diabetes (T2D) are associated with an increased risk of cardiovascular disease (CVD) [2,3,4]. CVD has been described as the leading cause of death in people with diabetes—accounting for up to 44% in T1D and up to 52% in T2D [2]. Early identification and risk stratification for higher risk of adverse CVD outcomes, with early interdisciplinary preventive multifactorial treatment, may reduce morbidity and mortality in people with T1D and T2D [5,6].

Diabetic heart disease encompasses an increased arteriosclerosis in the large arteries, such as the aorta, femoral arteries, and carotids, as well as microangiopathy, clinically apparent as neuropathy, retinopathy, and renal failure [1]. Additionally, diabetes mellitus increases the risk of heart failure independent of underlying conditions, such as coronary heart disease or arterial hypertension. The pathogenesis of the so-called diabetic cardiomyopathy (DCM) is multifactorial, whereby several hypotheses have been discussed previously, including autonomic dysfunction, interstitial fibrosis as well as metabolic and protein derangements [1,7]. Sustained hyperglycemic levels in diabetes mellitus may result in increased myocardial stiffness and impaired contractility [1,7,8,9]. The subsequent clinical appearance may be characterized by the presence of systolic dysfunction of the left ventricle (LV), as well as diastolic dysfunction including a reduction of the early diastolic filling of the LV, an increase in atrial filling, as well as an extension of the isovolumetric filling period of the LV [1,4,5,10,11,12,13].

Echocardiography for functional and morphological cardiovascular assessment in T1D and T2D is commonly performed in daily practice. Next to structural echocardiographic assessment, newer functional echocardiographic techniques, such as deformation pattern analysis based on speckle-tracking and tissue Doppler imaging (TDI), provide novel insights of regional and global myocardial performance including diastolic and systolic interaction assessment in diabetes [1,4,7,14,15,16,17]. However, recent studies revealed the clinical importance of subclinical systolic dysfunction assessed using speckle-tracking echocardiography as the first subclinical manifestation of DCM, appearing ahead of diastolic dysfunction and systolic impairment with subsequent overt heart failure [14,15,16,17,18,19,20,21]. These innovative novel approaches, focusing on functional echocardiographic assessment and remodeling, might provide a promising perspective for early DCM progression monitoring and treatment management, as well as prevention of overt DCM heart failure [17].

In this context, exercise-related dynamic cardiac remodeling analyzed by performing two-dimensional echocardiography, including deformation analysis of the LV global longitudinal strain (LV-GLS), the deformation pattern of the right ventricle (RV) and left atrium (LA) at rest as well, as post-peak performance in people with T1D are lacking. Therefore, the feasibility of dynamic cardiac remodeling of the right and left heart was investigated in eight asymptomatic participants with T1D to provide novel insights into the assessment of cardio-physiological adaption in T1D without overt heart failure. We hypothesized that there would be significant differences in the deformation analysis parameters of the left and right heart at rest and post peak exercise, revealing a subclinical impaired cardiocirculatory functional adaption and providing further insights into the characterization of asymptomatic DCM.

## 2. Materials and Methods

The study protocol was approved by the local ethics committee of the University of Bayreuth (O1305/1-GB, 22-020). Additionally, the study was registered at the German Clinical Trials Register (DRKS-ID DRKS00030738). The study was conducted in conformity with the declaration of Helsinki and Good Clinical Practice [22]. Participating subjects were informed about the study protocol and—before any study-related activities—they were asked to give their written informed consent.

### 2.1. Study Population

Eight cardiological-asymptomatic adults with T1D (5 males, 3 females), participating in the AppEx study, i.e., “Safety and pilot efficacy of a mobile app-based decision support system for exercise in Type 1 diabetes—the AppEx trial (DRKS00030738)”, were examined and evaluated at our research facility by an experienced cardiologist for specific dynamic cardiac remodeling of the right and left heart using two-dimensional echocardiography including speckle-tracking analysis after exhaustive cardiopulmonary exercise testing (CPET). All eight participating adults were vaccinated against coronavirus disease (COVID-19), and no participating T1D subject had to be excluded from the secondary outcome analysis of the AppEx study due to clinical apparent coronavirus disease (COVID-19) or post-COVID-19-infection syndromes.

Eligibility criteria included male or female subjects aged 18–65 years with a body mass index (BMI) of 18.0–29.9 kg/m^2^, both inclusive. Furthermore a clinically diagnosed T1D ≥ 12 months and being treated with multiple daily insulin injections ≥ 12 months, as well as a HbA1c level ≤ 10% (86 mmol/mol) were a prerequisite to be enrolled in the study. During CPET a mass-specific peak oxygen uptake > 20 mL/min/kg^−1^ served as a prerequisite for individual study enrollment. All participants were assessed for anthropometric data and two-dimensional transthoracic echocardiographic analyses, including strain analysis at rest and post peak exercise on a static bicycle. The obtained data of the subjects (*n* = 8; male *n* = 5, female *n* = 3) were compared for structural and functional echocardiographic parameters at rest and post peak exercise. No participants had a cardiological medical history, including symptomatic DCM, previous myocardial infarction, apparent severe heart failure, defined as > NYHA II level (New York Heart Association (NYHA)), and interventional or surgical revascularization, or exercise-related history for sudden cardiac death in all participating subjects and within their families.

### 2.2. Echocardiographic Examination

An echocardiographic functional and morphological assessment at rest and post-maximal-exercise testing on the bicycle was performed using a commercially available echocardiographic system Phillips EPIQ 7 device with an X5-1 Matrix-array transducer (Phillips Healthcare, Eindhoven, The Netherlands), following a standard protocol as previously described [23,24]. During individual echocardiography of the participants’ continuous heart rate data, assessment (in bpm) was performed. A two-dimensional echocardiographic assessment at resting conditions was performed according to general recommendations [23,24,25,26,27,28]. The systolic LV-EF assessment was performed using biplane Simpson rule, based on the apical two-chamber—as well as apical four-chamber view [24]. Left-atrial volume index (LAVI) and two-dimensional linear dimensions of the right and left heart were evaluated for both ventricles and both atria manually according to recent standards [23,24,26,28,29]. The systolic function of the RV was evaluated using the TAPSE (Tricuspid annular plane systolic excursion) at rest in the apical four-chamber view [24,28]. The specific assessment of the LV by calculating the LV mass index (LVMI in g/m^2^) and the relative wall thickness (RWT) was performed using the formula recommended by the current guidelines [24,27]. In evaluating the diastolic function of the LV the pulse-wave Doppler was measured in the apical four-chamber view referring to the peak early filling (E wave) and late diastolic filling (A wave) velocities, as described in previous research [24]. To assess the E/E′ratio in the participating subjects, the tissue Doppler of the lateral mitral annulus in the apical four-chamber view (peak early velocity E′) was assessed [23,24,27].

Focusing on the dynamic deformation pattern of the right and left heart, we recorded the LV, RV, and LA strain patterns of the T1D subjects´ hearts at resting conditions as well as ten minutes post-maximal bicycle CPET as a post-exercise assessment. Our secondary outcome analysis on dynamic cardiac remodeling in asymptomatic T1D focused on the left-heart global-longitudinal-strain pattern (LV-GLS) as well as on the right-heart deformation pattern, such as RV free-wall longitudinal deformation (RV FW long.Def.), as well as RV four-chamber longitudinal deformation (RV 4C long.Def.) [24]. Focusing on the dynamic LA pattern, our assessment was performed according to the recent European Association of Cardiovascular Imaging (EACVI) recommendations, as reported previously [24,30]. In this context, the phasic LA pattern assessment includes the specific analyses of LA reservoir strain (LASr), LA conduit strain (LAScd), and LA contraction strain (LASct) pattern [30]. Regarding our studied participants, no history of atrial fibrillation was known, which might have limited our LA strain assessment.

Furthermore, during the functional and morphological assessment no significant left- and right-heart valve regurgitation were detected, except mild clinical asymptomatic regurgitations as part of the previously described standard echocardiographic assessment [23,24,25]. 

### 2.3. Statistical Analyses

Statistical data analyses were performed with Graph Pad Prism 9 (Graph Pad Software; San Diego, CA, USA). Primarily, all data were tested for normal distribution via the Shapiro–Wilk test. Subsequently, data were tested for differences with paired *t*-tests, with statistical significance being accepted at *p* ≤ 0.05. All data are presented as mean ± SD.

## 3. Results

### 3.1. Baseline T1D Subjects’ Characteristics and Structural and Functional Echocardiographic Assessment at Resting Conditions

The anthropometric data and baseline characteristics of the participating male and female T1D subjects (*n* = 8, male *n* = 5, female *n* = 3) are presented in Table 1.

In the two-dimensional structural and functional echocardiographic assessment, all participants showed a normal systolic LV-EF at rest, estimated using the biplane Simpson method and a preserved dynamic contractile reserve of the LV post exercise. The structural assessment of the LV, the LVMI, and the RWT of the LV did not show any relevant pathological findings, and, with respect to LA and LV geometric assessment, no significant interindividual differences. Furthermore, the diastolic assessment of the LV did not indicate any pathological findings and, additionally, only mild clinically asymptomatic regurgitation at the tricuspid and mitral valves were revealed. None of the analyzed participants showed any relevant systolic pulmonary artery pressure using the tricuspid peak-systolic-velocity assessment. The obtained baseline echocardiographic characteristics can be categorized as within the normal range compared to data from sedentary control measurements by the German Society of Cardiology (DGK), as presented in previous research [24,28]. The obtained baseline echocardiographic characteristics are displayed in Table 2.

### 3.2. T1D Speckle-Tracking Analysis of the Left and Right Heart at Resting Conditions and Dynamic Functional Cardiac Remodeling Post Peak Exercise

Analyzing the dynamic functional cardiac remodeling, no significant differences were found for the RV free-wall longitudinal deformation (RV FW long.Def. rest −23.85 ± 6.40% vs. RV FW long.Def. post peak exercise −26.05 ± 6.92%, *p* = 0.5839) nor for the RV apical four-chamber longitudinal deformation (RV 4C long.Def. rest −20.58 ± 3.93% vs. RV 4C long.Def. post peak exercise −21.45 ± 4.77%, *p* = 0.7419) at rest and post peak exercise, as presented in Table 3.

The evaluation of the dynamic functional remodeling of the left heart revealed no significant differences for the LV-GLS pattern (*p* = 0.5952, as presented in Table 3), i.e., LV-GLS values at rest (−18.05 ± 2.31%) and slightly reduced values for LV-GLS post peak exercise (−17.41 ± 3.24%).

Analyzing the dynamic functional LA remodeling based on the average phasic LA strain (LAS) deformation pattern during all three phases of the atrial cycle, as presented in our previous research [24], we were not able to elucidate significant differences, neither for LASr analysis (*p* = 0.8379) nor for LAScd analysis (*p* = 0.0624, results represented in Table 3).

**Figure 1:**
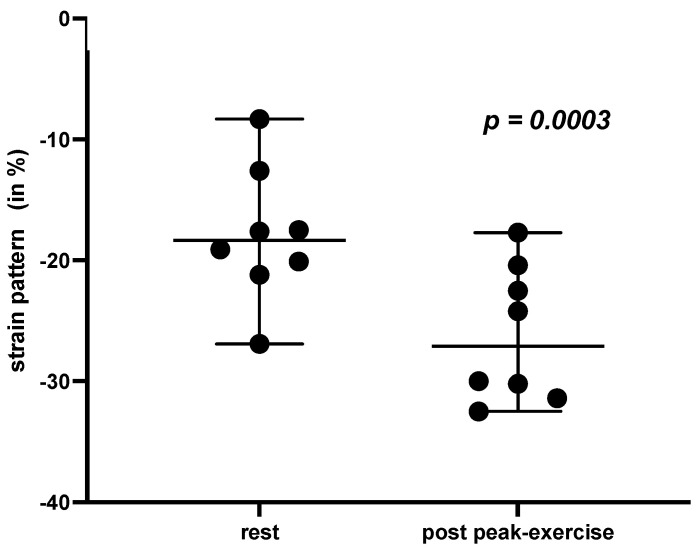
T1D—LA contractile strain pattern at rest and post peak exercise.

Significant differences across the participants could be elucidated for the LASct assessment at rest versus post peak-exercise parameters (LASct rest −17.91 ± 5.89% vs. LASct post peak exercise −26.11 ± 5.61%, *p* = 0.0003, as presented in Table 3 and Figure 1).

## 4. Discussion

The objective of this secondary outcome analysis was to assess right- and left-heart dynamic myocardial deformation patterns in cardiac-healthy individuals with T1D, using advanced speckle-tracking echocardiography at resting conditions and post peak exercise. Therefore, an early-stage identification of subclinical cardiac lesions in asymptomatic DCM, which might be characterized by functional and structural lesions and be involved in the progression to symptomatic heart failure, is essential for individual´s prognosis and therapeutic approach, and our innovative approach might contribute to primary assessment of subclinical cardiac lesions [16,31].

Previous research on the identification of early systolic LV-dysfunction in asymptomatic individuals with T1D demonstrated that decreased values of LV-GLS, defined as >−18.7%, are a common finding in asymptomatic T1D individuals without previous history of significant microvascular or macrovascular complications [32]. In this context, possible interacting factors, such as chronic hyperglycemia and increased adiposity have been reported previously [16,32]. Additionally, abnormal global peak longitudinal strain pattern of the LV have been reported for a certain association with elevated coronary calcium score (CACS) and visceral fat mass [16]. Our obtained results for slightly decreased LV GLS mean data at resting conditions (−18.05 ± 2.31) have to be interpreted while referring these preconditions. Nevertheless, our asymptomatic T1D individuals displayed preserved dynamic deformation strain pattern post peak exercise and no significant dynamic myocardial strain reduction. Previous research on echocardiographic changes during variable glycemic levels, especially acute hypoglycemia and post recovery, even hyperglycemic and euglycemic levels, revealed a significant impact on dynamic LV-EF and LV-GLS data [33]. According to our preserved dynamic LV-GLS mean data, the following conditions have to be taken into consideration: previously reported coherences between exercise-related hypoglycemic level during maximal CPET effort, high glycemic variability, and clinically inapparent cardiovascular disease [33]. Additionally, previous research revealed a detrimental effect of anterior-chest-wall deformities, such as most notably pectus excavatum, on cardiac motion and function. Therefore, an association between the degree of anterior-chest-wall deformity and myocardial strain magnitude was elucidated, whereby this anthropometric feature was not evaluated in our study population, as stated in the limitation section [34]. Our novel innovative dynamic “stress” approach might accompany the classical guideline-recommended risk-factor assessment and standard two-dimensional echocardiography in people with T1D without known heart disease to improve risk prediction for these patients at risk for cardiovascular disease [35]. The meaningfulness of dynamic stress myocardial deformation assessment might be regarded as an innovative prognostic tool with a high-clinical-negative predictive value in asymptomatic T1D. Due to the small number of participants, we were not able to correlate our obtained LV strain pattern data with diabetes duration and glycated hemoglobin (HbA1c) level, which are both associated with LV-GLS impairment [36].

Previously, RV evaluation in individuals with DCM was not frequently undertaken, whereby within the last decades its importance in the clinical course and prognosis of DCM has been recognized and has been focused scientifically [37]. Previous research revealed contrary findings with decreased RV myocardial performance in asymptomatic T1D compared to healthy matched controls at resting conditions [37], as well as preserved subclinical systolic function of the RV in early stage T1D subjects [38]. In our participating individuals, we could demonstrate preserved RV FW long.Def. and RV 4C long.Def. at rest without any significant exercise-related decrease, displaying preserved dynamic diastolic and systolic RV function post peak exercise. This novel dynamic RV myocardial assessment in asymptomatic T1D shows its feasibility and might contribute to better early-stage detection and understanding of subclinical abnormalities due to the beginning DCM focusing on the impact of RV performance.

Additionally, our dynamic deformation pattern analyses of asymptomatic people with T1D without any relevant previous cardiac history revealed significant differences for the LASct assessment at rest versus post peak-exercise parameters. This novel observation based on our feasibility research might provide novel insights into cardio-physiological adaption and early-stage risk stratification in asymptomatic T1D. Previous research revealed controversial findings, whereby in young healthy winter-sport professionals the dynamic data assessment of the left atrial cycle demonstrated significant differences for the conduit cycle of the LA strain analysis [24], and in people with cardiac amyloidosis and consecutive LV hypertrophy, atrial mechanics were significantly reduced with LA strain assessment in resting conditions [39]. In fact, LAS alterations are reported in several cardiovascular conditions before, whereby LA dysfunction is related to several predispositions, such as diastolic and systolic LV function, LV filling pressure, and functional and morphological LA structure [40,41,42]. In our participants, concomitant phenomena such as increased myocardial stiffness and impaired contractility [1,7,8,9], might contribute to the novel-revealed exercise-related LAS alterations, especially associated in the LASct phases. Investigations on this interesting topic are rare, and very little is known about the possible underlying pathophysiological determinants. It remains a matter for clarification whether the previously described predispositions, such as varying LV and LA properties, contribute to functional and morphological LAS modifications or whether the T1D individuals primarily have LA myopathy or the assessed LA deformation pattern are physiological due to post-exercise status [39]. The question that arises, of whether it is a T1D phenomenon or a normal post-exercise finding, remains unsolved in our explorative trial and has to be studied in further research with an appropriate matched control group. Even though our feasibility study might not point out any chronological causalities, our findings might be handled as hypothesis generating for subclinical LAS alterations in asymptomatic T1D. Assessment of these novel interesting strain deformation patterns might lead to early screening for DCM and may be worth investigation in larger prospective studies.

Our results are limited by several conditions: firstly, we performed a feasibility observational trial with a single-centre nature and a low number of participants, whereby further larger prospective studies should be initiated with appropriate statistical power analysis. In this regard, the main limitation of the study is that we do not provide a healthy matched control group and are solely discussing the obtained comparison cohort data pre and post exercise. Our previous research in elite winter sport athletes suggests comparable results, but do not represent an ideal comparison cohort for our participants with T1D. Secondly, the participating individuals displayed a certain inter-individual variability with respect to T1D duration and anthropometric data, especially the male participants. In this context, in our study population the possible influence of chest-wall conformation, such as narrow antero-posterior chest diameter, on cardiac kinetics and function at rest and post exercise has not been assessed in the participant´s anthropometric evaluation [34]. These preconditions should be given some attention interpreting our feasibility study data, and, therefore, our results have to be handled with caution. Our findings, based on dynamic deformation pattern assessment of the right and left heart in T1D, firstly elucidate and compare RV, phasic LA and LV strain alterations at resting conditions compared to post peak-exercise data. Our results might display a first explorative data assessment and need to be confirmed in larger prospective studies analyzing the clinical pathophysiological mechanisms and their transferability into clinical practice.

## 5. Conclusions

This explorative secondary outcome data assessment provides novel insights of preserved contractile reserve of the LV and elucidates dynamic modification of the phasic LA contractile deformation pattern in asymptomatic people with T1D post peak exercise.

In conclusion, dynamic deformation pattern analysis of the left and right heart is feasible in people with T1D and might contribute to better early-stage detection and understanding of subclinical abnormalities before established DCM. Further data are warranted to validate our obtained findings and “to characterize” a disease-specific dynamic deformation pattern of DCM with the aim of transferring it to everyday clinical practice.

## Figures and Tables

**Table 1:** Anthropometric and T1D characteristics.

	T1D Male *n* = 5	T1D Female *n* = 3
Age (years)	47.0 ± 17.5	39.3 ± 14.4
Height (cm)	179.2 ± 5.7	163.3 ± 6.4
Weight (kg)	76.7 ± 10.4	61.8 ± 0.8
BMI (kg/m^2^)	23.8 ± 2.6	23.2 ± 1.4
Duration of T1D (years)	16.8 ± 10.1	12.0 ± 1.7
HbA1c (%)	6.4 ± 0.3	7.8 ± 1.9

Data are presented as a median with standard deviation. Abbreviations: cm, centimeter; kg, kilogram; m^2^, square meter; T1D, Type 1 diabetes.

**Table 2:** Baseline echocardiographic characteristics.

	T1D Male *n* = 5	T1D Female *n* = 3	Reference Value Male	Reference Value Female
LV edd (mm)	48.00 ± 4.53	38.33 ± 2.52	42–58	38–52
LV Mass Index (g/m^2^)	79.40 ± 24.03	58.33 ± 5.51	49–115	43–95
Relative Wall Thickness RWT	0.37 ± 0.03	0.43 ± 0.04		
IVSd (mm)	9.40 ± 1.14	9.00 ± 0	6–10	6–9
LVPWd (mm)	8.80 ± 0.84	8.33 ± 0.58	6–10	6–9
E/A	1.62 ± 0.72	1.13 ± 0.06		
E/E’	7.76 ± 1.30	6.56 ± 2.20		
LAVI (mL/m^2^)	27.40 ± 6.54	30.33 ± 6.66		
LV − EF_rest_ (%)	59.40 ± 0.89	60.00 ± 5.00	52–72	54–72
LV − EF_post-stress_ (%)	69.40 ± 1.76	70.00 ± 5.00		

Data are presented as a median with standard deviation. Abbreviations: LV edd, left ventricle enddiastolic size; LV, left ventricular; IVSd, interventricular septal wall thickness at diastole; LVPWd, left ventricular posterior wall thickness at diastole; E/A, E/E’, parameters for diastolic function of left ventricle; LAVI, left atrial volume index; LV – EF, left ventricular systolic ejection fraction.

**Table 3:** Dynamic deformation pattern of LA, RV, and LV in T1D subjects.

	T1D Rest *n* = 8	T1D Post Exercise *n* = 8	*p*-Value
RV FW long Def.	−23.85 ± 6.40	−26.05 ± 6.92	0.5839
RV 4C long Def.	−20.58 ± 3.93	−21.45 ± 4.77	0.7419
LV GLS mean	−18.05 ± 2.31	−17.41 ± 3.24	0.5952
LV reservoir	41.45 ± 9.15	40.53 ± 7.99	0.8379
LA conduit	−23.53 ± 8.28	−16.15 ± 6.51	0.0624
LA contractile	−17.91 ± 5.89	−26.11 ± 5.61	0.0003 *

Data are presented as a median with standard deviation. * represents statistical significant results. Abbreviations: RV, right ventricle; FW, free wall; long, longitudinal; Def., deformation; 4C, four chamber; LV, left ventricle; GLS, global longitudinal strain; LA, left atrium.

## Data Availability

Individual anonymized data supporting the analyses of this study contained in this manuscript will be made available upon reasonable written request from researchers whom propose use of data for a specific purpose which has been approved.

## References

[1] Boudina S., Abel E.D. (2007). Diabetic Cardiomyopathy Revisited. Circulation.

[2] Morrish N.J., Wang S.-L., Stevens L.K., Fuller J.H., Keen H. (2001). Mortality and Causes of Death in the WHO Multinational Study of Vascular Disease in Diabetes. Diabetologia.

[3] Matteucci E., Giampietro O. (2014). Epidemiology of Cardiovascular Disease in Patients with Type 1 Diabetes: European Perspective. Exp. Clin. Endocrinol. Diabetes.

[4] Theilade S., Rossing P., Jensen J.S., Jensen M.T. (2018). Arterial-Ventricular Coupling in Type 1 Diabetes: Arterial Stiffness Is Associated with Impaired Global Longitudinal Strain in Type 1 Diabetes Patients—The Thousand & 1 Study. Acta Diabetol..

[5] Gæde P., Lund-Andersen H., Parving H.-H., Pedersen O. (2008). Effect of a Multifactorial Intervention on Mortality in Type 2 Diabetes. N. Engl. J. Med..

[6] Black J.A., Sharp S.J., Wareham N.J., Sandbæk A., Rutten G.E.H.M., Lauritzen T., Khunti K., Davies M.J., Borch-Johnsen K., Griffin S.J. (2014). Does Early Intensive Multifactorial Therapy Reduce Modelled Cardiovascular Risk in Individuals with Screen-detected Diabetes? Results from the ADDITION-Europe Cluster Randomized Trial. Diabet. Med..

[7] Spector K.S. (1998). Diabetic Cardiomyopathy. Clin. Cardiol..

[8] Berg T.J., Snorgaard O., Faber J., Torjesen P.A., Hildebrandt P., Mehlsen J., Hanssen K.F. (1999). Serum Levels of Advanced Glycation End Products Are Associated with Left Ventricular Diastolic Function in Patients with Type 1 Diabetes. Diabetes Care.

[9] Avendano G.F., Agarwal R.K., Bashey R.I., Lyons M.M., Soni B.J., Jyothirmayi G.N., Regan T.J. (1999). Effects of Glucose Intolerance on Myocardial Function and Collagen-Linked Glycation. Diabetes.

[10] Persson M., Östling G., Smith G., Hamrefors V., Melander O., Hedblad B., Engström G. (2014). Soluble Urokinase Plasminogen Activator Receptor. Stroke.

[11] Llauradó G., Ceperuelo-Mallafré V., Vilardell C., Simó R., Gil P., Cano A., Vendrell J., González-Clemente J.-M. (2014). Advanced Glycation End Products Are Associated with Arterial Stiffness in Type 1 Diabetes. J. Endocrinol..

[12] Forbes J.M., Cooper M.E. (2013). Mechanisms of Diabetic Complications. Physiol. Rev..

[13] Aso Y., Inukai T., Tayama K., Takemura Y. (2000). Serum Concentrations of Advanced Glycation Endproducts Are Associated with the Development of Atherosclerosis as Well as Diabetic Microangiopathy in Patients with Type 2 Diabetes. Acta Diabetol..

[14] Mochizuki Y., Tanaka H., Matsumoto K., Sano H., Shimoura H., Ooka J., Sawa T., Motoji Y., Ryo-Koriyama K., Hirota Y. (2017). Impact of Left Ventricular Longitudinal Functional Mechanics on the Progression of Diastolic Function in Diabetes Mellitus. Int. J. Cardiovasc. Imaging.

[15] Jensen M.T., Sogaard P., Andersen H.U., Bech J., Fritz Hansen T., Biering-Sørensen T., Jørgensen P.G., Galatius S., Madsen J.K., Rossing P. (2015). Global Longitudinal Strain Is Not Impaired in Type 1 Diabetes Patients Without Albuminuria. JACC Cardiovasc. Imaging.

[16] Van Berendoncks A.M., Van Gaal L., De Block C., Buys D., Salgado R., Vrints C., Shivalkar B. (2019). Abnormal Longitudinal Peak Systolic Strain in Asymptomatic Patients with Type I Diabetes Mellitus. Echocardiography.

[17] Minciună I., Hilda Orășan O., Minciună I., Lazar A., Sitar-Tăut A.V., Oltean M., Tomoaia R., Puiu M., Sitar-Tăut D., Pop D. (2021). Assessment of Subclinical Diabetic Cardiomyopathy by Speckle-tracking Imaging. Eur. J. Clin. Invest..

[18] Berceanu M., Mirea O., Donoiu I., Militaru C., Săftoiu A., Istrătoaie O. (2020). Myocardial Function Assessed by Multi-Layered Two-Dimensional Speckle Tracking Analysis in Asymptomatic Young Subjects with Diabetes Mellitus Type 1. Cardiology.

[19] Cameli M., Mandoli G.E., Lisi E., Ibrahim A., Incampo E., Buccoliero G., Rizzo C., Devito F., Ciccone M.M., Mondillo S. (2019). Left Atrial, Ventricular and Atrio-Ventricular Strain in Patients with Subclinical Heart Dysfunction. Int. J. Cardiovasc. Imaging.

[20] Tadic M., Cuspidi C. (2021). Left Atrial Function in Diabetes: Does It Help?. Acta Diabetol..

[21] Ifuku M., Takahashi K., Hosono Y., Iso T., Ishikawa A., Haruna H., Takubo N., Komiya K., Kurita M., Ikeda F. (2021). Left Atrial Dysfunction and Stiffness in Pediatric and Adult Patients with Type 1 Diabetes Mellitus Assessed with Speckle Tracking Echocardiography. Pediatr. Diabetes.

[22] Harriss D.J., MacSween A., Atkinson G. (2019). Ethical Standards in Sport and Exercise Science Research: 2020 Update. Int. J. Sports Med..

[23] Evangelista A., Flachskampf F., Lancellotti P., Badano L., Aguilar R., Monaghan M., Zamorano J., Nihoyannopoulos P. (2008). European Association of Echocardiography Recommendations for Standardization of Performance, Digital Storage and Reporting of Echocardiographic Studies. Eur. J. Echocardiogr..

[24] Zimmermann P., Eckstein M.L., Moser O., Schöffl I., Zimmermann L., Schöffl V. (2022). Left Ventricular, Left Atrial and Right Ventricular Strain Modifications after Maximal Exercise in Elite Ski-Mountaineering Athletes: A Feasibility Speckle Tracking Study. Int. J. Environ. Res. Public Health.

[25] Zimmermann P., Moser O., Eckstein M.L., Wüstenfeld J., Schöffl V., Zimmermann L., Braun M., Schöffl I. (2021). Athlete’s Heart in Elite Biathlon, Nordic Cross—Country and Ski-Mountaineering Athletes: Cardiac Adaptions Determined Using Echocardiographic Data. J. Cardiovasc. Dev. Dis..

[26] Lang R., Bierig M., Devereux R., Flachskampf F., Foster E., Pellikka P., Picard M., Roman M., Seward J., Shanewise J. (2006). Recommendations for Chamber Quantification☆. Eur. J. Echocardiogr..

[27] Lang R.M., Badano L.P., Mor-Avi V., Afilalo J., Armstrong A., Ernande L., Flachskampf F.A., Foster E., Goldstein S.A., Kuznetsova T. (2015). Recommendations for Cardiac Chamber Quantification by Echocardiography in Adults: An Update from the American Society of Echocardiography and the European Association of Cardiovascular Imaging. J. Am. Soc. Echocardiogr..

[28] Hagendorff A., Fehske W., Flachskampf F.A., Helfen A., Kreidel F., Kruck S., la Rosée K., Tiemann K., Voigt J.-U., von Bardeleben R.S. (2020). Manual Zur Indikation Und Durchführung Der Echokardiographie—Update 2020 Der Deutschen Gesellschaft Für Kardiologie. Kardiologe.

[29] Galderisi M., Cosyns B., Edvardsen T., Cardim N., Delgado V., di Salvo G., Donal E., Sade L.E., Ernande L., Garbi M. (2017). Standardization of Adult Transthoracic Echocardiography Reporting in Agreement with Recent Chamber Quantification, Diastolic Function, and Heart Valve Disease Recommendations: An Expert Consensus Document of the European Association of Cardiovascular Imaging. Eur. Heart J. Cardiovasc. Imaging.

[30] Badano L.P., Kolias T.J., Muraru D., Abraham T.P., Aurigemma G., Edvardsen T., D’Hooge J., Donal E., Fraser A.G., Marwick T. (2018). Standardization of Left Atrial, Right Ventricular, and Right Atrial Deformation Imaging Using Two-Dimensional Speckle Tracking Echocardiography: A Consensus Document of the EACVI/ASE/Industry Task Force to Standardize Deformation Imaging. Eur. Heart J. Cardiovasc. Imaging.

[31] Weber T.R., da Silva R.L., Cossul S., Alves M.S.L., Lee S.V.d.S., Marques J.L.B. (2021). The Use of Echocardiography in Type 1 Diabetes. Rev. Port. Cardiol..

[32] Kapelios C.J., Bonou M., Barmpagianni A., Tentolouris A., Tsilingiris D., Eleftheriadou I., Skouloudi M., Kanellopoulos P.N., Lambadiari V., Masoura C. (2021). Early Left Ventricular Systolic Dysfunction in Asymptomatic Patients with Type 1 Diabetes: A Single-Center, Pilot Study. J. Diabetes Complicat..

[33] Andreasen C.R., Andersen A., Hagelqvist P.G., Lauritsen J.V., Jørgensen P.G., Engberg S., Faber J., Hartmann B., Pedersen-Bjergaard U., Knop F.K. (2022). Hypoglycaemia and Rebound Hyperglycaemia Increase Left Ventricular Systolic Function in Patients with Type 1 Diabetes. Diabetes Obes. Metab..

[34] Sonaglioni A., Nicolosi G.L., Trevisan R., Lombardo M., Grasso E., Gensini G.F., Ambrosio G. (2023). The Influence of Pectus Excavatum on Cardiac Kinetics and Function in Otherwise Healthy Individuals: A Systematic Review. Int. J. Cardiol..

[35] Jensen M.T., Sogaard P., Gustafsson I., Bech J., Hansen T.F., Almdal T., Theilade S., Biering-Sørensen T., Jørgensen P.G., Galatius S. (2019). Echocardiography Improves Prediction of Major Adverse Cardiovascular Events in a Population with Type 1 Diabetes and without Known Heart Disease: The Thousand & 1 Study. Diabetologia.

[36] Zairi I., Mzoughi K., Kamoun S., Moussa F.B., Rezgallah R., Maatoug J., Mazigh S., Kraiem S. (2019). Impairment of Left and Right Ventricular Longitudinal Strain in Asymptomatic Children with Type 1 Diabetes. Indian Heart J..

[37] Ahmed T.A., Ahmed Y.A., Arafa A.I., Salah R.A. (2018). Detection of Occult Right Ventricular Dysfunction in Young Egyptians with Type 1 Diabetes Mellitus by Two-Dimensional Speckle Tracking Echocardiography. Indian Heart J..

[38] Berceanu M., Mirea O., Târtea G.C., Donoiu I., Militaru C., Istrătoaie O., Săftoiu A. (2019). The Significance of Right Ventricle in Young Subjects with Diabetes Mellitus Type 1. An Echocardiographyic Study. Curr. Health Sci. J..

[39] Brand A., Frumkin D., Hübscher A., Dreger H., Stangl K., Baldenhofer G., Knebel F. (2021). Phasic Left Atrial Strain Analysis to Discriminate Cardiac Amyloidosis in Patients with Unclear Thick Heart Pathology. Eur. Heart J. Cardiovasc. Imaging.

[40] Ersbøll M., Andersen M.J., Valeur N., Mogensen U.M., Waziri H., Møller J.E., Hassager C., Søgaard P., Køber L. (2013). The Prognostic Value of Left Atrial Peak Reservoir Strain in Acute Myocardial Infarction Is Dependent on Left Ventricular Longitudinal Function and Left Atrial Size. Circ. Cardiovasc. Imaging.

[41] Miglioranza M.H., Badano L.P., Mihăilă S., Peluso D., Cucchini U., Soriani N., Iliceto S., Muraru D. (2016). Physiologic Determinants of Left Atrial Longitudinal Strain: A Two-Dimensional Speckle-Tracking and Three-Dimensional Echocardiographic Study in Healthy Volunteers. J. Am. Soc. Echocardiogr..

[42] Santos A.B.S., Roca G.Q., Claggett B., Sweitzer N.K., Shah S.J., Anand I.S., Fang J.C., Zile M.R., Pitt B., Solomon S.D. (2016). Prognostic Relevance of Left Atrial Dysfunction in Heart Failure With Preserved Ejection Fraction. Circ. Heart Fail..

